# A draft genome of field pennycress (*Thlaspi arvense*) provides tools for the domestication of a new winter biofuel crop

**DOI:** 10.1093/dnares/dsu045

**Published:** 2015-01-27

**Authors:** Kevin M. Dorn, Johnathon D. Fankhauser, Donald L. Wyse, M. David Marks

**Affiliations:** 1 Department of Plant Biology, University of Minnesota, Saint Paul, MN 55108, USA; 2 Department of Agronomy and Plant Genetics, University of Minnesota, Saint Paul, MN 55108, USA

**Keywords:** field pennycress, *Thlaspi arvense*, *de novo* assembly, whole genome sequencing, comparative genomics

## Abstract

Field pennycress (*Thlaspi arvense* L.) is being domesticated as a new winter cover crop and biofuel species for the Midwestern United States that can be double-cropped between corn and soybeans. A genome sequence will enable the use of new technologies to make improvements in pennycress. To generate a draft genome, a hybrid sequencing approach was used to generate 47 Gb of DNA sequencing reads from both the Illumina and PacBio platforms. These reads were used to assemble 6,768 genomic scaffolds. The draft genome was annotated using the MAKER pipeline, which identified 27,390 predicted protein-coding genes, with almost all of these predicted peptides having significant sequence similarity to Arabidopsis proteins. A comprehensive analysis of pennycress gene homologues involved in glucosinolate biosynthesis, metabolism, and transport pathways revealed high sequence conservation compared with other Brassicaceae species, and helps validate the assembly of the pennycress gene space in this draft genome. Additional comparative genomic analyses indicate that the knowledge gained from years of basic Brassicaceae research will serve as a powerful tool for identifying gene targets whose manipulation can be predicted to result in improvements for pennycress.

## 1. Introduction

Next-generation sequencing (NGS) has enabled the characterization and comparison of whole genomes for a growing list of plant species.^1–5^ This same technology is being used to speed up and facilitate the breeding of crop plants.^3,6–8^ NGS also has the potential to enable new approaches to domesticate crops.^9^ One species targeted for domestication is *Thlaspi arvense* (field pennycress, pennycress herein). Pennycress is a member of the Brassicaceae in the tribe Thlaspideae native to Eastern Europe.^10,11^ Pennycress plants are diploid, propagate largely via self-fertilization, and have a 1C DNA content of 539 Mb.^12^ Naturalized populations are distributed worldwide, including North America, South America, and Australia.^10,11^ Previous molecular studies showed that the original *Thlaspi* genus was polyphylogenic, and several members of the *Thlaspi* genus were kept in a monophylogenic Thlaspideae tribe.^13^ *Thlaspi caerulescens*, which is a well-studied heavy metal accumulator,^14,15^ was moved to the genus *Noccaea* during this reorganization.^13,16^ Two members of the original *Thlaspi* genus, *Thlaspi ceratocarpum* and *Thlaspi alliaceum* (roadside pennycress), are in the Thlaspideae along with *Alliaria petiolata* (garlic mustard) and *Peltaria alliacea* (garlic cress) and several other species. The Brassicaceae are divided into three lineages, and the members of the Thlaspideae are in lineage II, which includes the *Brassica* genus, but not *Arabidopsis* (lineage I).^17^ Similar to *Brassica napus* and *Brassica rapa*, pennycress produces seeds with high oil content (30% by weight).^18^

Field pennycress is being developed as a new winter cover crop and biodiesel feedstock for the Midwestern United States that can be seeded into standing corn.^9,19–21^ As a winter annual, pennycress germinates in the fall and overwinters as a vegetative rosette. It exhibits extreme winter hardiness, surviving the harsh winters that are common to the Canadian Plains and Midwestern United States.^10^ Pennycress flowers and matures early in the spring, producing up to 1,300 kg/hectare of seed that can be harvested in time for planting an additional summer annual crop of soybeans.^9,22^ The oils found in pennycress seed are suitable for biodiesel production,^21^ and the remaining seed meal is high in protein that could serve as an additional revenue source for farmers.^11^ As a winter cover, pennycress would provide important ecosystem services. In much of the Midwest, the ground lies barren from late fall until early summer, which can increase nutrient runoff and soil erosion.^23^ The addition of a fall-planted pennycress cover crop should reduce nutrient leaching and erosion, which would help sustain current farming practices. In the United States alone, over 16 million hectares currently used in the corn to soybean rotation could be used for pennycress production without displacing current food production systems.^20,24^

As a weedy species, there are many challenges that will need to be addressed in order to convert pennycress into a new crop species.^9^ Pennycress already has many natural attributes such as the high seed yield and oil content described above. However, earlier maturing varieties are needed to ensure that pennycress can be harvested early, allowing for full-season soybean production. Seed dormancy also is an issue,^25,26^ as is common in many weed species; we have observed highly variable germination rates in preliminary field trials. Pennycress oil and protein meal are not currently suitable for animal or human consumption.^27^ Making pennycress seeds edible would add value to the crop. In addition, the fatty acids contained in the oil are adequate for conversion to biodiesel, but can be further optimized.^18,21^ It is unclear whether traditional breeding programmes can address these concerns, as there may be insufficient natural variation in wild populations.

*Arabidopsis thaliana* has been used as a key organism to address questions concerning plant development for the past 30 yrs.^28^ Arabidopsis research has resulted in an understanding of many plant developmental processes, such as the control of time to flower, of seed maturation, and of seed oil synthesis.^29–31^ During the course of these studies, many mutations have been identified in Arabidopsis that confer what can be considered agronomically desirable phenotypes.^9^ The function of pennycress genes can be predicted on the basis of their similarity to genes with known function in Arabidopsis.^9^ With this information, it should be possible to identify mutations that confer desirable traits in pennycress using mutation-based breeding tools such as TILLING,^32^ DeTILLING,^33^ and CRISPR-Cas.^34^ To enable the use of these technologies and to take advantage of the information derived from research on Arabidopsis and other Brassica species, a genomic sequence is needed for pennycress.

To generate a draft genome for pennycress, a natural population of plants was isolated from a roadside near Coates, Minnesota called MN106. Little is known about natural pennycress populations at the genome sequence level. In this report, we have examined this population at the genome level using several different NGS strategies to *de novo* assemble a draft genome. This assembly captures the vast majority of pennycress gene space as predicted by comparison with other Brassica species. The draft genome detailing the pennycress gene space has allowed the function of pennycress genes to be predicted on the basis of their similarity to genes with known function in Arabidopsis. In addition, the analysis revealed that even in mixed natural populations, the selfing mode of reproduction maintains individual plants in a highly homozygous state.

## 2. Materials and methods

### 2.1. Plant materials

*Thlaspi arvense* line MN106 has been previously described and originates from Coates, MN.^19^ Seed from a MN106 bulk planting was germinated on moist Berger BM2 germination mix (Berger Inc., www.berger.ca), stratified at 4°C for 7 days, and grown in climate-controlled growth chambers at the University of Minnesota (21°C, 16 h/8 h day/night cycles at 100 μmol/m^2^/s PAR). Individual plants were transplanted to 4-inch pots 2 weeks after germination. Six-week-old plants with established rosettes were vernalized at 4°C for 30 days in the dark. After vernalization, plants were returned to growth chambers. In all, nine plants were selected for DNA extraction. After tissue harvest, the same nine plants were maintained, and seeds were individually collected from each plant.

### 2.2. Genomic DNA isolation and DNA sequencing

To enrich the total amount of nuclear DNA sequenced, nuclei were purified from young leaf tissue using a series of density gradient centrifugation steps using an adapted protocol.^35^ The full protocol for genomic DNA isolation is listed in the Supplementary Methods. Illumina sequencing (100 bp paired-end library run on Illumina HiSeq 2000, 250 bp paired-end library run on Illumina MiSeq, 50 bp long-jump mate pair libraries run on Illumina HiSeq 2000) was completed at the University of Minnesota Genomics Center. Pacific Biosciences (PacBio RSII) sequencing was completed at the Mayo Clinic Molecular Biology Core (Rochester, MN). All raw sequencing files have been submitted to NCBI Sequence Read Archive under accession number SRP033211. FASTQ files from all sequencing runs were imported and subjected to quality control using the Sequencing QC Report tool in CLC Genomics Workbench Version 6.5 (CLC Bio, www.clcbio.com). Details on DNA sequencing library preparation and quality control parameters are described in the Supplementary Methods.

### 2.3. Genome assembly, scaffolding, and annotation

The pennycress draft genome was assembled and annotated using two desktop computers built specifically for this purpose. The components and specifications of these computers are listed in Supplementary Table S1. Genome sequencing reads were trimmed and *de novo* assembled in CLC Genomics Workbench Version 6.5 (CLC Bio, www.clcbio.com). The CLC assembler has previously been used in assembling complex plant genomes, such as the barley (*Hordeum vulgare*)^36^ and rubber tree (*Hevea brasiliensis*)^37^ genome projects. Additionally, the CLC assembler has a low Random Access Memory (RAM) requirement, opposed to other popular *de Bruijn* graph assemblers that can require hundreds of gigabytes of RAM. Initial assembled scaffolds over 1000 bp from the CLC assembly were scaffolded using SSPACE,^38^ and remaining gaps were filled using GAPFILLER.^39^ The genome assembly was annotated using the MAKER pipeline.^40^ A full description of assembly and annotation parameters is available in Supplementary Methods.

### 2.4. Comparative genomics

Comparative studies on the structural arrangement and synteny of the pennycress genome assembly were performed using SynMap (www.genomeevolution.org/CoGe/). To examine the synteny between the *Eutrema salsugineum* and *Thlaspi arvense* genomes, seven pseudochromosome sequences were constructed by concatenating the corresponding genomic scaffolds.^1^ *Thlaspi arvense* genomic scaffolds >75 kb in length were compared with the seven *E. salsugineum* pseudochromosomes using a Syntenic Path Assembly in SynMap (DAGChainer – Relative Gene order, −D = 20, −A = 5, skip random/unknown chromosomes). BLASTp comparisons of the 27,390 predicted pennycress peptides were performed in CLC Genomics Workbench using the predicted peptide databases for *A. thaliana*,^41^ *Arabidopsis lyrata*,^42^ *B. rapa*,^43^ *Capsella rubella*,^5^ and *E. salsugineum*^1^ using the following BLAST parameters: Expectation Value = 10, Word Size = 7, Filter Low Complexity = Yes, Protein Matrix = BLOSUM62, Gap Existence = 11, Gap Extension = 1*.* Peptide sequences for *A. thaliana* were obtained from ftp://ftp.arabidopsis.org/home/tair/Proteins/TAIR10_protein_lists/TAIR10_pep_20101214. Sequences for the remaining species were obtained from Phytozome (www.phytozome.net). BLASTn analyses of the previously published pennycress transcriptome^19^ against the genome assembly were performed using the following parameters: Match Cost = 2, Mismatch Cost = 3, Gap Existence = 2, Gap Extension = 2, Expectation Value = 10, Filter Low Complexity = Yes, Maximum Number of Hits = 15.

### 2.5. Read mapping and variant detection for cleaved amplified polymorphic sequence marker design

Trimmed and filtered sequencing reads from the Illumina HiSeq 2000 100 bp paired-end experiment were re-mapped to the genome assembly to identify potential heterozygosity or variation in the MN106 accession sequenced. Reads were mapped to the assembly in CLC Genomics Workbench using the ‘Map Reads to Reference’ tool (masking mode = no masking, mismatch cost = 3, insertion cost = 3, deletion cost = 3, length fraction = 0.95, similarity fraction = 0.95, global alignment = no, auto detect paired distances = yes, non-specific match handling = map randomly) and variants called using the Probabilistic Variant Detection tool (ignore non-specific matches = yes, ignore broken pairs = yes, minimum coverage = 10, variant probability = 90, require both forward and reverse reads = no, maximum expected variants = 2, ignore quality scores = no). Sites declared as single-nucleotide polymorphisms (SNPs) were manually examined to identify cleaved amplified polymorphic sequence (CAPS) markers in which one variant was a member of a six base recognition site for a DNA restriction endonuclease, and the other variant resulted in the loss of the restriction site. Four sites were identified, and primers were designed to amplify these regions for CAPS analysis (Supplementary Table S4). As a control, the primers were designed to flank both a CAPS site and a conserved restriction site that would be cleaved in both variants. DNA was isolated using the Mag-Bind EZ Plant DNA Kit (Omega BioTek, www.omegabiotek.com). Polymerase chain reactions were performed using Q5 High Fidelity DNA Polymerase (New England Biolabs, www.neb.com) with these DNA extracts and the corresponding CAPS primers to confirm the potential variants.

### 2.6. Comparative analysis of genes involved in glucosinolate metabolism and transport

*A. thaliana*, *B. rapa*, and *Brassica oleraceae* genes previously identified to be involved in glucosinolate biosynthesis, breakdown, and transport were derived from a previous study.^4^ Alignments of predicted peptide sequences for each gene were performed in CLC Genomics Workbench using the Create Alignment tool (Gap open cost = 10, Gap extension cost = 1, End gap cost = Free, Alignment mode = Very accurate). Neighbour Joining trees were created in CLC Genomics Workbench (Protein distance measure = Jukes-Cantor, Perform bootstrap analysis = Yes, Replicates = 100). To examine expression levels of predicted gene models, Illumina RNAseq reads from the previously published transcriptome assembly^19^ were trimmed and filtered (Illumina TruSeq Trim Adaptor 3, Ambiguous Trim = Yes, Ambiguous Limit = 2, Quality Trim = Yes, Quality Limit = 0.05, Also search on reversed sequence = Yes, Remove 5′ terminal nucleotides = Yes, Number of 5′ terminal nucleotides to remove = 10, Remove 3′ terminal nucleotides = no, Save broken pairs = Yes), and mapped to the annotated draft genome (Mapping Type = Map to gene regions only, Maximum number of hits for a read = 10, Strand Specific = Both, Count paired read as two = Yes, Expression Value = RPKM, Reference type = Genome annotated with genes and transcripts, Global alignment = no, Auto detect paired distances = Yes, Similarity fraction = 0.8, Length Fraction = 0.8, Mismatch cost = 2, Insertion cost = 3, Deletion cost = 3).

## 3. Results and discussion

### 3.1. Genome sequencing and assembly

Pennycress is a diploid species with a haploid number of seven chromosomes and a genome size of 539 Mb.^12^ In order to isolate a sufficient quantity of nuclear DNA for the various sequencing libraries used, nuclear DNA was isolated from nine plants derived from an MN106 isolate that had been maintained in the lab for several generations. This DNA was sequenced using both the Illumina and PacBio platforms, generating over 47 Gb of sequencing data representing over 87× coverage of the predicted genome size (Table 1). Illumina HiSeq 2000 and MiSeq reads were *de novo* assembled using the *de Bruijn* graph-based CLC Genomics Workbench assembler. PacBio reads were not used to create the *de Bruijn* graph, but instead they were only used to resolve ambiguities during the graph building stage.

**Table 1.** Genome sequencing, assembly, and annotation statistics

Library type	Number of reads	No. of nucleotides after QC (Mb)
*Thlaspi arvense* genome sequencing		
Illumina HiSeq 2000 (2 × 100 bp paired end)	352,394,426	33,190.50
Illumina MiSeq (2 × 250 bp paired end)	6,291,688 (merged)	2,589.33
	8,548,686 (unmerged)	1,904.90
Illumina HiSeq 2000 (2 × 50 bp Mate Pair) 2, 3, 5 kb inserts	209,815,249	9,550.66
PacBio SMRT cell (four cells)	110,751	214.78
	Scaffolds	Contigs in scaffolds
*T. arvense* genome assembly		
Number	6,768	44,109
Mean length (bp)	50,681	7,375
N50 (bp)	140,815	21,096
Total assembly length (bp)	343,012,389	325,295,785
*T. arvense* genome annotation		
Number of gene models	27,390	
Gene models highly similar to TAIR10 peptide (*e* < 1 × 10^−5^ and >70% positive per cent)	85.94%	
GC content	37.99%	
Repetitive DNA sequence	24.38%	
Retroelements (20.94%)	78,812	
DNA transposons (1.6%)	12,382	

Summary statistics for DNA sequencing libraries, genome assembly and scaffolding, and genome annotation.

The initial CLC assembly resulted in 206,726 initial scaffolds ≥200 bp, encompassing 392,190,998 bp. Of this assembly, initial scaffolds over 1,000 bp (*n* = 50,064, 322,949,692 bp total length) were further joined using long-insert Illumina mate pair reads in SSPACE, and remaining gaps were filled using GAPFILLER. This analysis resulted in the formation of 6,768 final scaffolds that encompass over 343 Mb with an average scaffold length of 50,681 bp and N50 value of 140,815 bp (Table 1). Over 60% of the assembled scaffolds are over 10,000 bp long, with 902 scaffolds over 100,000 bp and 9 scaffolds over 1,000,000 bp (Supplementary Table S2). The longest 3,000 scaffolds represented over 85% of the assembly length (Supplementary Fig. S1). The 156,662 small scaffolds (<1000 bp) from the initial CLC assembly that were excluded from the final scaffolding and gap-filling manipulations encompassed 69,241,306 bp (Supplementary Table 2). These small initial scaffolds were excluded from further analyses as they likely represent sequences from repetitive regions of the genome, which are difficult to assemble and were unlikely to contribute to gene identification efforts. These small initial scaffolds represent a significant portion (12.8%) of the predicted genome size. With the addition of the initial small scaffolds, the total assembled length of the draft genome presented here is 412,253,695 bp, 76.5% of the predicted pennycress genome size of 539 Mb.

As the draft genome is incomplete (<80% of the predicted genome size) and fragmented, developing a more complete and contiguous assembly will be important for both plant improvement efforts and answering basic questions about the genomics and evolution of pennycress. However, the reported contig N50 of 21 kb is well within the norm of other recently reported genome assemblies based on Illumina reads such as those for *Aquilaria agallocha*—14.6 kb,^44^ *Sesamum indium*—52.2 kb,^2^ *Citrullus lantus*—26.4 kb,^45^ and *Cicer arietinum*—23.54 kb.^6^ The sequence of contigs directly reflects the sequenced gene space, which is especially important for a species like pennycress where this information enables one to identify genes of interest whose manipulation via either overexpression or knockdown can be predicted to confer agronomically desirable phenotypes.

### 3.2. Genome annotation with MAKER

Genome annotation using the MAKER pipeline^40^ annotated 27,390 predicted protein-coding genes with an average total length of 2,195 bp, average coding sequence length of 1,238 bp, and an average of 5.541 exons/gene (Supplementary Dataset S2). More than 89% of the predicted gene models are supported by at least 5 RNA sequencing (RNAseq) reads from the previously published *de novo* transcriptome assembly, while 1452 gene models (5.3%) lack RNAseq read support (Supplementary Dataset S2). Over 85% of the predicted peptides (23,538) have at least one highly significant BLASTp hit (*e* < 1 × 10^−5^ and >70% positive percent) to an *A. thaliana* (TAIR10) predicted peptide (Table 1). Another 1,876 predicted peptides (6.8%) show a significant hit (e < 1 × 10^−5^), but at a lower positive percent value (>60%). Only 173 predicted peptides lacking an *A.thaliana* BLASTp hit were found (Expectation value >10) (Supplementary Dataset S2). BLASTn analyses of the 33,873 *de novo* assembled contigs from the pennycress transcriptome^19^ against the draft genome indicate a high level of completeness of the predicted gene space. Over 88% of transcripts (30,053) had BLASTn hits (≥95% identity and *e* ≤ 1 × 10^−5^) in the genome, while 95.8% of assembled transcripts (32,458) had a significant hit (*e* ≤ 1 × 10^−5^) but at a lower percent identity threshold (>75%) (Supplementary Dataset S3). Only 167 transcriptome contigs lacked a BLASTn hit in the genome. (Expectation value >10) A separate BLASTn analysis of these 167 transcripts against the genomes of *A. thaliana*,^41^ *A. lyrata*,^42^ *B. rapa*,^43^ *C. rubella*,^5^ and *E. salsugineum*^1^ revealed that 40 transcripts lacked any match to these five species, while the remaining 127 consisted of low quality and short hits, likely indicative of misassembled sequences from the *de novo* assembled transcriptome (Supplementary Dataset S4). The repetitive DNA content of the final pennycress genome scaffolds was assessed by RepeatMasker.^46^ It was found that repetitive elements constituted 24% of the draft genome. This analysis identified 78,812 retroelements encompassing >71 Mb of the assembly, consisting mainly of Gypsy/DIRS1-type, long-terminal repeats (60.3 Mb). 12,382 DNA transposons representing >5 Mb of the draft genome were also found, including hobo-Activator (0.8 Mb) and Tourist/Harbinger (0.9 Mb) type elements (Supplementary Table S3). Both the raw sequencing reads and assembled sequences have been submitted to NCBI, which has been supplemented by a pennycress genome database containing a JBrowse genome browser,^47^ BLAST database, and data repository available at pennycress.umn.edu.

### 3.3. Comparative genomics of the pennycress genome assembly

**Figure 1.**
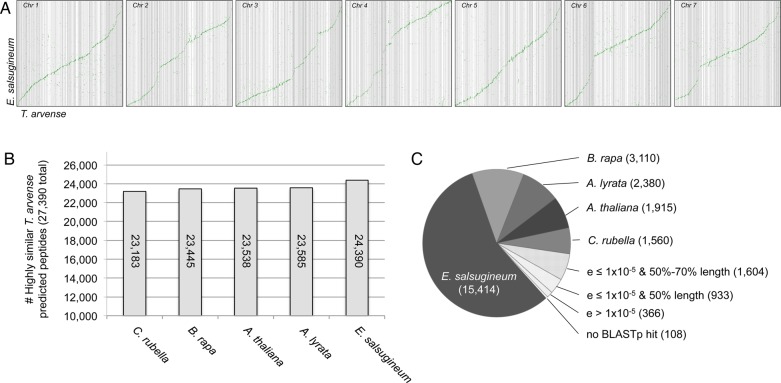
Comparative genomics of pennycress and other Brassicaceae species. (A) Syntenic path assembly dot plots comparing pennycress scaffolds >75 kilobases long to the seven *Eutrema salsugineum* pseudochromosomes fromYang *et al.*^1^ (B) BLASTp analysis of the 27,390 predicted pennycress peptides against predicted peptide sets from *Capsella rubella*,^5^ *Brassica rapa*,^43^ *Arabidopsis thaliana* (Bevan and Initiative, 2000), *Arabidopsis lyrata*,^42^ and *Eutrema salsugineum*. Highly similar is defined as pennycress predicted peptide having at least one BLASTp hit *e* < 1 × 10^−5^ and positive sequence similarity >70%. (C) BLASTp analysis of predicted pennycress peptides against a protein database containing the predicted peptides of the five Brassicaceae species listed. Predicted peptides with top hits (*e* ≤ 1 × 10^−5^ and >70% hit length) to a predicted protein from the corresponding species are shown, with pennycress peptides with hits falling below this threshold shown in the lower right half of the pie chart.

Of the sequenced Brassicaceae genomes, pennycress is most closely related to *E. salsugineum*, which possesses a much smaller genome (241 Mb), but the same karyotype (*n* = 7).^1,48^ To evaluate the relative completeness of the genome assembly, we used a syntenic path assembly comparison of the pennycress assembly to the *E. salsugineum* reference genome.^1^ Pennycress genomic scaffolds >75 kb long, representing 241 Mb (>70%) of the assembly were compared with the 241 Mb reference genome of *E. salsugineum*. Large portions of the seven *E. salsugineum* pseudochromosomes possess a high degree of synteny with the pennycress assembly, indicative of the close evolutionary relationship between these two species, as well as a high level of completeness of conserved regions in the pennycress genome (Fig. 1A).

To evaluate the quality of the genome assembly and predicted gene models, a combination of comparative analyses were used to compare the draft pennycress genome with the gene models of *A. thaliana*, *A. lyrata*, *B. rapa*, *C. rubella*, and *E. salsugineum*. BLASTp analyses of the 27,390 predicted pennycress peptides against five separate databases containing these five Brassicaceae species revealed that over 23,000 of the pennycress peptides had highly similar hits in all five species, with *E. salsugineum* possessing the highest proportion (>89%) of highly similar predicted peptides (Fig. 1B; Supplementary Dataset S5). Similarly, in a BLASTp analysis of the pennycress predicted peptides against a single database containing all five Brassicaceae species, 15,414 predicted pennycress peptides had highly similar (*e* ≤ 1 × 10^−5^ and ≥70% hit length) hits to *E. salsugineum* (Supplementary Dataset S6). An additional 8,965 predicted peptides had highly similar hits to *B. rapa*, *A. lyrata*, *A. thaliana*, and *C. rubella,* while 2,903 BLASTp hits fell outside of these parameters, and 108 of the predicted pennycress peptides lacked a BLASTp hit (Fig. 1C).

### 3.4. Evaluation of zygosity in the sequenced population

The draft genome was constructed using DNA isolated from nine plants that were several generations removed from a single population first identified in Coates, MN. A variant detection analysis was performed to assess the degree of homozygosity among the individual plants used for sequencing (see Materials and Methods for parameters used in this analysis). We detected 131,906 SNPs, which is ∼1 SNP per 2.6 kb. Given the high level of sequencing coverage and stringent quality control, it is unlikely that the predicted SNPs were solely due to sequencing errors. We developed three hypotheses to explain the SNPs. First, these SNPs represent inappropriately assembled duplicated regions of the pennycress genome. In this analysis, if the first hypothesis were true and the declared SNPs were artificial, then we would not expect any differences between the nine different plants. Second, the SNPs may represent evidence of heterozygosity throughout the genome that would occur if the plants were prone to a high degree of outcrossing. If the second hypothesis were true, then we would expect that the nine plants would segregate at ∼1 : 1 : 2 for the homozygosity of the presence or absence of a SNP or would be heterozygous with one chromatid containing the site and the other lacking the site, respectively. Third, distinct highly homozygous populations made up the original collection of plants used to isolate DNA that was sequenced. In this case, the genomes of any individual in the population would be expected to be largely homozygous at any particular locus.

**Figure 2.**
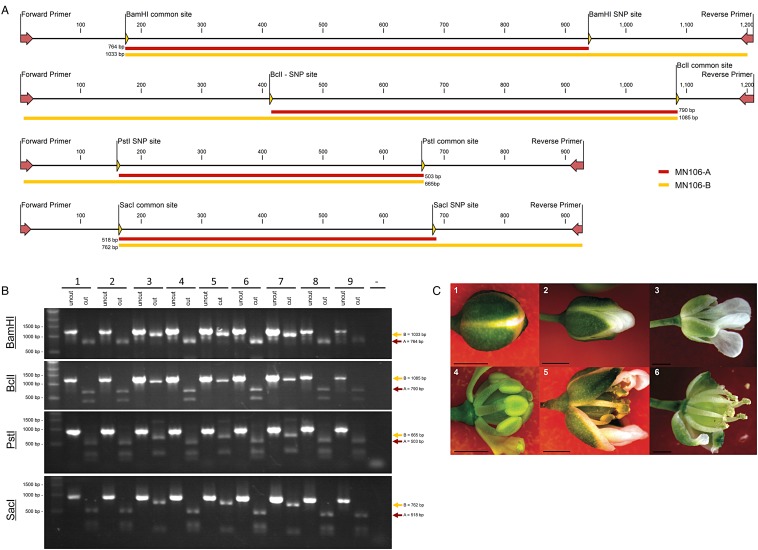
CAPS analysis of *Thlaspi arve*nse line MN106 (A) Schematic of the four PCR fragments produced by the primer sets listed in Supplementary Table S4. The largest fragments used to distinguish between individuals containing the SNP (MN106 A genotype fragment—top, and MN106 B genotype fragment—bottom). (B) DNA was isolated from progeny of each of the nine plants used to produce the draft genome assembly, and analysed using four CAPS markers. PCR products for each plant are shown side-by-side undigested (uncut) and post-digestion (cut) with the corresponding restriction endonucleases. In all cases, samples 3, 5, and 7 share restriction digest patterns, corresponding to the MN106-B genotype. A negative control for the PCR reaction is shown in the last lane. (C) Morphology of developing *T. arvense* flowers. The top panel (1–3) shows the morphology of the unaltered flowers, while the bottom panel (4–6) shows the same series of flowers with sepals and petals either removed or rearranged to reveal the status of the stamens with regard to filament elongation and the shedding of pollen. (4) Neither filament elongation nor pollen shedding has commenced in (1). (5) Filaments have elongated, and pollen is being shed inside of the closed flower shown in (2). (5) Pollen densely covers the stigmatic surface by the time the flower is fully open in (6). All scale bars equal 1 mm.

CAPS^49^ analysis using DNA isolated from progeny of the individual plants that were used to generate the draft genome was performed to distinguish between these three hypotheses. Primer sequences used to amplify regions used in the CAPS analysis shown in Fig. 2A are listed in Supplementary Table S4. Individuals were shown to either distinctly contain or lack the variant at four restriction enzyme sites, which eliminated the first hypothesis that these SNPs represented divergence in paralogous genes or misassembly of duplicated regions. Furthermore, none of the samples showed evidence of heterozygosity. Plants 3, 5, and 7 lacked the cut sites at the polymorphic regions, and plants 1, 2, 4, 6, 8, and 9 were homozygous for the cut sites (Fig. 2B). This supports the third hypothesis that the original MN106 population contained at least two distinct, highly homogenous populations. The fact that three individuals lacked all the cut sites and six individuals contained all the cut sites is likely due to the fact that at every CAPS locus, one prominent variant was detected in the variant detection analysis. Loci with the prominent variant that contained the six base restriction site were chosen for the CAPS analysis.

Based on previous analyses, a high degree of homozygosity among wild pennycress individuals was not unexpected, as the cleistogamous development of pennycress flowers (pollination occurring inside a closed flower) minimizes outcrossing (Fig. 2C).^11^ These findings will aid pennycress breeding programmes, as progeny from crosses will be expected to readily self-pollinate with minimal need for isolation. In addition, the analysis provides an extra level of validation by showing that a subset of the identified SNPs are real and not created by sequencing or assembly errors. These SNPs have the potential to be used in breeding and geographical studies, and are available in Supplementary Dataset S7. These results suggest that the original MN106 population consists of at least two distinct haplotypes on the basis of the CAPS analysis. This suggests that even in mixed populations, the selfing nature of pennycress reduces heterozygosity. This is an important finding, as it will facilitate the isolation and generation of highly inbred populations.

### 3.5. Analysis of genes involved in glucosinolate metabolism

To explore the functionality of our assembly, we determined how well the predicted gene space accounted for genes involved in glucosinolate biosynthesis. Glucosinolates (GSLs) are a diverse class of secondary metabolites common throughout the Brassicaceae that are important for plant/pathogen and plant/herbivore interactions.^50^ The underlying genetic mechanisms controlling the biosynthesis, transport, and breakdown of GSLs within the Brassicaceae have largely been dissected, with many of the genes responsible for this process having been characterized.^51^ Upon biosynthesis of the core glucosinolate structure (derived from one of several amino acids), GSLs generally lack bioactivity until plant tissue is damaged, leading to the hydrolysis of the GSL structure to one of several by-products. This breakdown process is mediated by enzymes called myrosinases, resulting in an unstable aglycone.^51^ The diversification of the final glucosinolate breakdown by-product is mediated by several classes of specifier proteins.^52^

Historically, pennycress has been characterized by its unique ‘garlic-like’ aroma that has been attributed to high levels of allylthiocyanate,^53^ which is a major hydrolysis by-product of the major GSL in above-ground pennycress tissues: allylglucosinolate.^54^ The high level of GSLs in pennycress gives rise to another common name of the species, stinkweed.^11^ The high level of GSLs and GSL by-products in pennycress is of significant agronomic and economic interest. Animal feed containing pennycress seed has traditionally been considered undesirable due to the high levels of GSLs in pennycress.^11^ However, after seed is pressed for oil, the remaining seed meal remains high in protein^55,56^ and presents a potential new source of billions of kilograms per year of high protein meal. If varieties with low GSL levels in seed can be developed, there is a potential for using defatted pennycress meal as an animal feed supplement.

**Figure 3.**
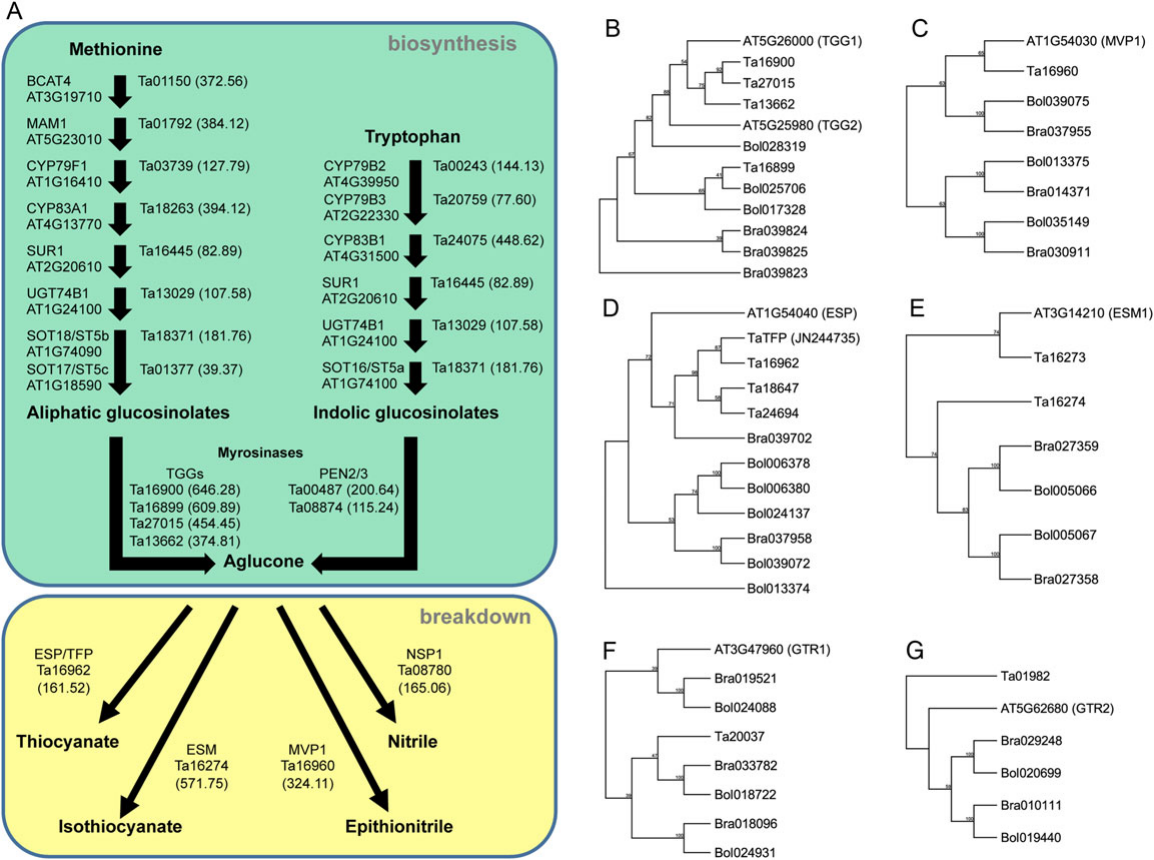
Analysis of genes involved in glucosinolate metabolism and transport. (A) Overview of glucosinolate biosynthesis core structure (top) via methionine and tryptophan and breakdown (bottom) and corresponding orthologues in the pennycress genome pathway derived from Liu *et al.*^4^ Expression values (RPKM, in parentheses) are shown for each putative orthologues derived from the global RNAseq reads previously described.^19^ (B–G) Neighbour joining trees of TGG1/TGG2, MVP1, ESP, ESM1, GTR1, and GTR2-like predicted peptides (100 bootstrap replicates) from pennycress (identified in this study), *Brassica rapa,* and *Brassica oleraceae*.^4^

BLASTp analyses were used to identify putative orthologues to known glucosinolate genes in Arabidopsis (Supplementary Dataset S2). Genes involved in the GSL core biosynthesis and breakdown pathway were derived from *A. thaliana*, *B. rapa*, and *B. oleraceae*.^4^ Putative orthologues were identified for GSL biosynthesis from methionine and tryptophan (Fig. 3A), along with several putative myrosinases (thioglucoside glucohydrolase—TGGs and atypical myrosinases—PEN2 and PEN3). Potential orthologues to several interesting specifier proteins were also identified (Fig. 3A—top). To obtain a semi-quantitative estimate of these predicted genes, RNAseq reads from the previously described transcriptome were used to obtain rough expression values for each gene model (Supplementary Dataset S1). As these RNAseq reads represent a global library representing various tissues, this analysis provides an initial probe into the pennycress genes potentially responsible for the unique glucosinolate composition of pennycress. For example, two putative myrosinases (Ta16900 and Ta16899) represent the 120th and 129th most highly expressed gene models, respectively (Fig. 3A and B; Supplementary Dataset S1). A modified vacuole phenotype1 (*MVP1*)-like pennycress gene was also identified (Ta16960—Fig. 3A). MVP1 in Arabidopsis interacts with the myrosinase TGG2 to modulate myrosinase activity.^57^

Potential orthologues to many important specifier proteins were also identified in this analysis and help explain the unique GSL activity in pennycress. For example, epithiospecifier modifier1 (ESM1) in Arabidopsis represses the formation of nitriles and favours isothiocyanate production.^58^ An *ESM1*-like pennycress gene, Ta16274 (Fig. 3A), was among the top 200 expressed genes models and could explain the high levels of isothiocyanate in pennycress.^54^ Additionally, another potential hydrolysis product of GSLs, epithionitriles, can only be formed from GSLs possessing terminal double bonds in their side chain (allylglucosinolate, for example). As allylglucosinolate represents over 90% of GSLs in above-ground tissue,^54^ this *ESM1*-like pennycress gene could represent a key GSL specifier protein also responsible for epithionitrile production.

The evolution of the identified specifier proteins was also examined. It has been previously reported that GSL-related genes commonly exist in tandemly duplicated regions of the genome within the Brassicaceae.^4,59^ The predicted peptides of *A. thaliana* TGG1 (Fig. 3B), MVP1 (Fig. 3C), ESP (Fig. 3D), ESM1 (Fig. 3E), GTR1 (Fig. 3F), and GTR2 (Fig. 3G) and corresponding orthologues in pennycress, *B. rapa*, and *B. oleraceae* were compared to determine sequence similarity and retention of tandemly duplicated regions throughout the genome. We identified three putative pennycress myrosinases with high sequence similarity to the Arabidopsis myrosinases TGG1 (AT5G26000) (Fig. 3B), with an additional predicted peptide (Ta16899) being more similar to *B. oleraceae* TGG2-like peptides (Bol9025706 and Bol017328). Only a single MVP1-like pennycress predicted peptide was identified in the draft genome (Fig. 3C). Three orthologous predicted peptides for AtESP (AT1G54040) were identified (Fig. 3D). The previously described TaTFP,^54^ which was cloned from a cDNA library, had a top BLASTp hit to Ta16961. However, the Ta16961 predicted peptide is nearly twice as long as the TaTFP predicted peptide (NCBI Accession JN244735) and appears to indicate either the TaTFP cDNA represented a unique splice variant or an erroneous splice site prediction from the annotation pipeline. An adjacent gene model, Ta16962, also had high similarity to TaTFP (Fig. 3D). Two tandem *ESM1*-like genes were also identified, similar to the tandemly duplicated orthologues in *B. rapa* (Bra027358 and Bra027359) and *B. oleraceae* (Bol005066 and Bol005067) (Fig. 3E).

Several orthologues to important GSL transport genes were also identified. The GSL transporters glucosinolate transporter (GTR) 1 and 2 have been shown to serve as major transporters responsible for the loading of GSLs into developing seeds in Arabidopsis, and loss-of-function mutations in these genes result in significantly reduced levels of GSLs in seeds.^60,61^ The identification of putative GTR1 and GTR2 orthologues (Fig. 3F) represents important targets for improvement in our ongoing breeding programme.

### 3.6. Identification of predicted orthologues of Arabidopsis genes that confer desirable phenotypes when mutated

There are several important traits that will need to be addressed during the domestication process to make pennycress a viable crop, such as reducing seed dormancy and increasing rates of flowering. In Arabidopsis, complete or partial loss of function mutations in a number of genes can confer agronomically desirable traits such as increased seed size, improved seed oil composition, earlier flowering and reduced seed dormancy, seed glucosinolate content, and seed pod shatter (Table 2). Here we show that predicted orthologues of these genes are present in our draft assembly (Table 2). While this is not an exhaustive list of potential gene targets, this represents a number of well-characterized genes responsible for controlling these phenotypes. Mutations in these genes in Arabidopsis can confer what would be considered agronomically desirable phenotypes. For example, pennycress GTR1- and GTR2-predicted orthologues are highly conserved at the predicted peptide level (88.5 and 94.8% positive match, respectively). Additionally, identifying natural or induced mutations in key regulatory genes like *DOG1*^62,63^ could lead to reduced levels of seed dormancy in pennycress. Similarly, developing winter annual lines that flower and mature rapidly in the spring should be possible through targeting genes involved in the vernalization and photoperiodic flowering time pathways, such as *FLC* and *FRI*^19^ (Table 2; Supplementary Dataset S8). The development of rapidly maturing lines will be particularly important in the upper Midwestern United States, as the shorter spring growing seasons at northern latitudes could limit the widespread adaptation of pennycress as a winter cover crop. By targeting specific important agronomic traits, and the underlying genetic mechanisms controlling these traits through the approaches mentioned above, we are poised to rapidly convert a wild weed species into a new highly valuable and sustainable winter oilseed crop.

**Table 2.** Putative orthologues controlling important agronomic traits in pennycress

Trait of interest	Arabidopsis gene	AGI no.	Corresponding pennycress predicted peptide ID	Pennycress genome scaffold no.	Arabidopsis peptide length	Pennycress peptide length	% Identity	% Positive
Seed dormancy	*DOG1*	AT5G45830	Ta16411	141	291	284	72.9	80.9
	*ABI3*	AT3G24650	Ta24539	630	720	728	86.8	90.6
	*ABI4*	AT2G40220	Ta07356	85	328	395	59.3	66.3
	*ABI5*	AT2G36270	Ta25088	828	442	434	85.8	89
Seed size	*WRI1*	AT3G54320	Ta07949	20	438	441	80.2	83.4
	*DGAT1*	AT3G51520	Ta05453	12	314	566	67.7	70.1
	*IKU2*	AT3G19700	Ta01151	3	991	1016	79.7	86.8
	*KLU*	AT1G13710	Ta14711	213	517	488	84.8	88.2
	*GL2*	AT1G79840	Ta08884	25	776	798	82.6	86
	*MUM4*	AT1G53500	Ta23332	457	667	597	83.6	85.8
Seed GSL content	*HAG1*	AT3G54610	Ta07928	20	568	592	85.8	88.3
	*GTR1*	AT3G47960	Ta20037	206	636	651	81	88.5
	*GTR2*	AT5G62680	Ta01982	4	616	613	90.3	94.8
Erucic acid content	*FAE1*	AT4G34520	Ta11742^a^	45	506	506	86.4	91.5
Linoleic/Linolenic acid content	*FAD2*	AT3G12120	Ta12495	59	383	404	84.3	88.2
Seed pod shatter	*SHP1*	AT3G58780	Ta15094	22	273	248	86.8	89.4
	*SHP2*	AT2G42830	Ta08438	22	248	290	76.6	80.7
	*IND*	AT4G00120	Ta25465	1003	198	172	61.5	69
	*ALC*	AT5G67110	Ta02444	6	210	207	72.8	77.9
	*RPL*	AT5G02030	Ta15425	92	575	639	80.5	83.3
	*FUL*	AT5G60910	Ta01807	57	242	208	63.3	68.1
Time to flower	*FLC*	AT5G10140	Ta00917	1	196	203	84.3	89.7
	*FRI*	AT4G00650	Ta26225^a^	1344	314	367	55	65.8

Identification of pennycress genes with high sequence similarity to *Arabidopsis* genes controlling key traits of interest, including genomic location and predicted peptide similarity.

^a^MAKER-derived gene models contained errors and were manually corrected to obtain predicted peptide sequence.

### 3.7. The future of genomics-based pennycress improvement

While the annotated draft genome for pennycress presented in this report will provide new tools for the domestication of a new winter oilseed crop, there is still much work to be done to develop a broad base of genomic resources for pennycress. For example, the creation of a complete, anchored reference genome is needed. The increasing length and quality of DNA sequencing reads from third-generation (single molecule—PacBio or Oxford Nanopore) sequencing technologies and new library preparation techniques such as Illumina TruSeq Synthetic Long-Reads (LRseq, formerly Moleculo)^64^ will allow for the *de novo* assembly of highly contiguous yet complex genomes. Combined with our ongoing development of a high-density genetic map using restriction site-associated DNA sequencing (RADseq),^65,66^ the anchoring and ordering of a highly contiguous genome assembly to the genetic linkage map should be on the immediate horizon to produce a complete reference genome for pennycress. With a completed pennycress genome, the phylogenetic resolution of the Brassicaceae expanded lineage 2 can be improved, including providing tools for the phylogenetic resolution of *Thlaspi* species,^67^ and answering questions on pennycress genome structure. The draft pennycress genome presented here will enable the development of a genomics-based breeding programme. In addition, the identification of the gene space will allow the use of techniques such as mutation breeding, TILLING of ethyl methanesulfonate-mutagenized populations, DeTILLING of fast neutron-mutagenized populations, and genomic selection. The use of precise genome editing techniques such as CRISPR/Cas9 and TALENs should also be on the horizon, as pennycress can be transformed using the *Agrobacterium*-mediated vacuum infiltration floral dip method (John Sedbrook, personal communication). Pennycress has the potential to be planted on over 16 million hectares in the United States alone and produce over 22 billion litres of oil suitable as a biodiesel feedstock.^20,21,24^ The successful domestication of pennycress has the potential to benefit farmers and the environment, provide a new source of biofuel, and reduce greenhouse gases associated with global climate change. The genomics-based domestication of pennycress represents an exciting example of the development of a new crop species.

## Conflict of interest statement

Any opinions, findings, and conclusions or recommendations expressed in this material are those of the authors, and do not necessarily reflect the views of the US National Science Foundation.

## Supplementary data

Supplementary Data are available at www.dnaresearch.oxfordjournals.org.

## Funding

This work was funded by the UMN Institute on the Environment, UMN CBS, and UMN College of Food, Agriculture, and Natural Resource Sciences, the Minnesota Agricultural Experiment Station, a DOE/USDA Plant Feedstock Genomics for Bioenergy program grant to MDM (USDA 2014-67009-22305), and by US National Science Foundation Graduate Research Fellowships to K.M.D. (00006595) and J.D.F. Funding to pay the Open Access publication charges for this article was provided by a DOE/USDA Plant Feedstock Genomics for Bioenergy program grant to MDM (USDA 2014-67009-22305).

## Data deposition

The Illumina and Pacific Biosciences sequencing reads for *Thlaspi arvense* line MN106 are available in the NCBI Sequence Read Archive under the accession number SRP033211. This Whole Genome Shotgun project has been deposited at DDBJ/EMBL/GenBank under the accession number AZNP00000000. The version described in this paper is version AZNP01000000. The genome assembly and related annotation files can also be downloaded from pennycress.umn.edu.

**Table:** 

	Accession	Run
*Thlaspi arvense* line MN106—Illumina 100 bp paired end	SRX380646	SRR1034657
*Thlaspi arvense* line MN106—Illumina 250 bp paired end	SRX380649	SRR1034659
*Thlaspi arvense* line MN106—Illumina 50 bp mate pair (2 kb insert)	SRX381531	SRR1035703
*Thlaspi arvense* line MN106—Illumina 50 bp mate pair (3.5 kb insert)	SRX381541	SRR1035705
*Thlaspi arvense* line MN106—Illumina 50 bp mate pair (7 kb insert)	SRX381551	SRR1035715
*Thlaspi arvense* line MN106—PacBio RSII 10 kb insert reads	SRX380881	SRR1035588

## Supplementary Material

Supplementary Data
