# Supplementary material for: A draft genome of field pennycress (Thlaspi arvense) provides tools for the domestication of a new winter biofuel crop
Source: DNA Res. 2015 Jan 27;22(2):121–31. doi: 10.1093/dnares/dsu045 (PMC4401323; doi:10.1093/dnares/dsu045)
Supplement: Supplementary Data [file supp_22_2_121__index.html]

A draft genome of field pennycress (Thlaspi arvense) provides tools for the domestication of a new winter biofuel crop — Supplementary Data 

# A draft genome of field pennycress (*Thlaspi arvense*) provides tools for the domestication of a new winter biofuel crop

## Supplementary Data

Supplementary Data

**Files in this Data Supplement:**

- Supplementary Figure 1 - pdf file
- Supplementary Table 1 - pdf file
- Supplementary Table 2 - pdf file
- Supplementary Data - Doc file
- Supplementary Dataset1 - xlsx file
- Supplementary Dataset2 - xlsx file
- Supplementary Dataset3 - xls file
- Supplementary Dataset4 - xlsx file
- Supplementary Dataset5 - xlsx file
- Supplementary Dataset6 - xlsx file
- Supplementary Dataset7 - xlsx file
- Supplementary Dataset8 - txt file
- Supplementary Table 3 - xls file
- Supplementary Table 4 - xls file
