# Supplementary material for: A draft genome of field pennycress (Thlaspi arvense) provides tools for the domestication of a new winter biofuel crop
Source: DNA Res. 2015 Jan 27;22(2):121–31. doi: 10.1093/dnares/dsu045 (PMC4401323; doi:10.1093/dnares/dsu045)
Supplement: Supplementary Data [file supp_dsu045_dsu045supp_fig1.pdf]

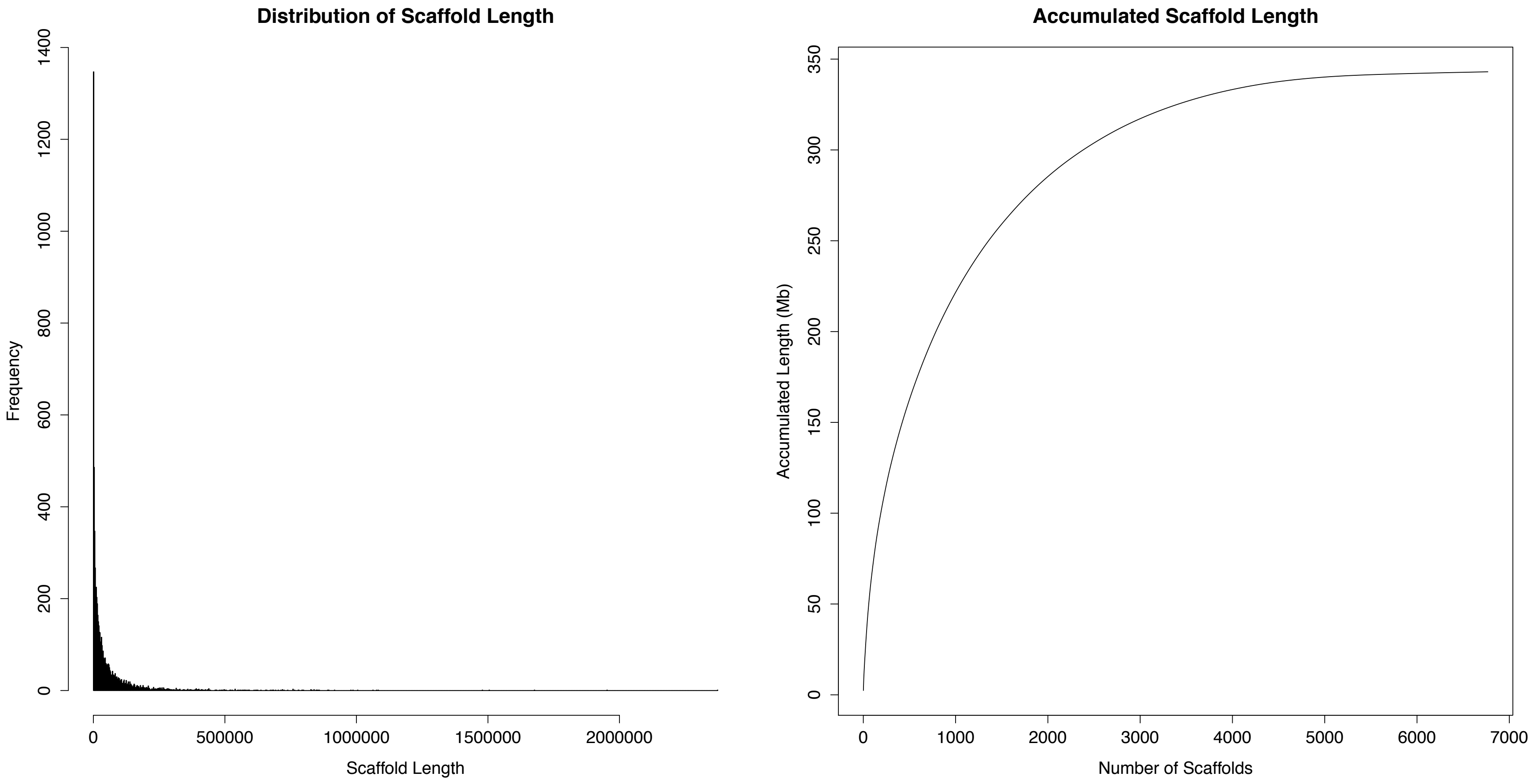

Figure S1: Distribution and Accumulated Length of Genomic Scaffolds
a.) Distribution of *de novo* assembled *Thlaspi arvense* genomic scaffolds.
b.) Accumulated length of genomic scaffolds. Over 85% of the total assembly length (>343 Mb) consists of the 3,000 longest scaffolds.
