# Supplementary material for: A draft genome of field pennycress (Thlaspi arvense) provides tools for the domestication of a new winter biofuel crop
Source: DNA Res. 2015 Jan 27;22(2):121–31. doi: 10.1093/dnares/dsu045 (PMC4401323; doi:10.1093/dnares/dsu045)
Supplement: Supplementary Data [file supp_dsu045_dsu045supp_table1.pdf]

Desktop computer components and assembly. The computer used for the pennycress genome assembly was configured and assembled using the components listed in Supplementary Table S1-A that were obtained from online retailers, which runs the Windows 7 operating system. The components used to build the desktop computer used for genome annotation are listed in Supplementary Table S1-B, which runs the Linux (BioLinux- http://environmentalomics.org/bio-linux/) operating system.

Supplementary Table S1-A: Components of Computer Used in Genome Assembly.

Component	Model and Manufacturer
CPU	Intel Core i7-3820 (Intel)
Motherboard	Asus Sabertooth X79 motherboard (Asustek Computer Inc.)
RAM	(2) G.SKILL Ripjaws Z Series 32 GB (4x8GB) Memory – Model F3-12800CL10Q-32GBZL (G.SKILL)
CPU Cooler	Cooler Master Hyper 212 EVO RR-212E-20PK-R2 CPU Cooler (Cooler Master)
Boot Hard Drive	256 GB Crucial M4 CT256M4SSD2 Solid State Hard Drive (Crucial)
Main Hard Drive	2 TB Western Digital WD Black WD2002FAEX Hard Drive (Western Digital)
SSD Caching	128 GB Samsung 840 Pro Series MZ-7PD128BW Solid State Hard Drive (Samsung)
Power Supply	Corsair Enthusiast Series TX650 V2 650W Power Supply
Video Card	EVGA 02G-P4-3653-KR GeForce GTX 650 Ti 2GB PCIe3.0 Video Card (EVGA)
Optical Drive	LITE-ON iHDS118-04 DVD/CD-ROM
Case	Corsair Carbide Series 500R Computer Case

Supplementary Table S1-B: Components Used in Computer for Genome Annotation.

Component	Model and Manufacturer
CPU	(2) Opteron 6344, 12-core 2.6ghz (AMD)
Motherboard	KGPE-D16 SSI EEB Server Motherboard (Asus)
RAM	(4 x 16GB) ValueRAM ECC Registered KVR16R11D4K4 (Kingston)
CPU Cooler	4-Dual Heat Pipe SSO Bearing CPU Cooler - NH-U9DO (Noctua)
Boot Hard Drive	500 GB - 840 Series Solid State Hard Drive - MZ-7TD500BW (Samsung)
Main Hard Drive	Barracuda 2 TB Hard Drive - ST2000DM001 (Seagate)
