# Supplementary material for: A draft genome of field pennycress (Thlaspi arvense) provides tools for the domestication of a new winter biofuel crop
Source: DNA Res. 2015 Jan 27;22(2):121–31. doi: 10.1093/dnares/dsu045 (PMC4401323; doi:10.1093/dnares/dsu045)
Supplement: Supplementary Data [file supp_dsu045_dsu045supp_table2.pdf]

**Supplementary Table S2**	
**Complete genome assembly statistics**	
**v1.0 pennycress genome assembly scaffolds**	
**Scaffold Statistics**	Value
Number of Scaffolds	6,768
Cumulative Scaffold Length	343,012,389
Number of Scaffolds >1000 bp	6,731
Number of Scaffolds >10,000 bp	4,099
Number of Scaffolds >100,000 bp	902
Number of Scaffolds >1,000,000	9
Mean Scaffold Size	50,681
Median Scaffold Size	17,246
N50 Scaffold Size	140,815
L50 Scaffold Count	561
Average number of contigs per scaffold	4
Average length of breaks (>25 Ns) between contigs in scaffolds	807
%N's in scaffolds	5.17%
**Scaffolded contig statistics**	
Number of Contigs in final scaffolds	44,109
Cumulative Contig Length (bp)	25,295,785
Number of Contigs >1000 bp	38,572
Number of Contigs >10,000 bp	7,953
Number of Contigs >100,000 bp	238
Mean Contig Size	7,375
Median Contig Size	2,458
N50 Contig Size	21,096
L50 Contig Count	3,513
**Small initial scaffold statistics (scaffolds <1000bp from initial CLC assembly)**	
Number of scaffolds	156662
Cummulative Scaffold Length	69241306
Longest scaffold	999
Shortest Scaffold	200
Mean Scaffold Size	442
Median Scaffold Size	386
N50 Scaffold Size	505
%N's	0.43%
