# Supplementary material for: A draft genome of field pennycress (Thlaspi arvense) provides tools for the domestication of a new winter biofuel crop
Source: DNA Res. 2015 Jan 27;22(2):121–31. doi: 10.1093/dnares/dsu045 (PMC4401323; doi:10.1093/dnares/dsu045)
Supplement: Supplementary Data [file supp_dsu045_dsu045supp.doc]

*S1. Detailed protocol for genomic DNA isolation*

All centrifugation steps were performed at 4°C. A total of 15 grams of young leaves were washed in cold, sterile ddH_2_O and homogenized in 300ml of 4°C nuclear isolation buffer (1 M sucrose, 10 mM Tris HCl (pH 7.2), 5 mM MgCl_2_, 10 mM 2-mercaptoethanol) in a Brinkmann Polytron homogenizer. Homogenized tissue was filtered through four layers of cheesecloth and one layer of Miracloth (EMD Millipore, [www.emdmillipore.com](http://www.emdmillipore.com/)) two times. Filtered lysate was centrifuged for 15 minutes at 9,500 RPM in a Beckman JA14 rotor at 4°C. The supernatant was discarded and the resulting pellet was resuspended in 50 ml of nuclear isolation buffer plus 250 μl of Triton X-100. The resuspended pellet was then twice centrifuged for 10 minutes at 9,500 RPM in a Beckman JA20 rotor, resuspending the pellet as above. The final pellet was resuspended in 50 ml of nuclear isolation buffer and placed onto a Percoll density gradient consisting of 60% and 35% layers (1x Percoll Buffer - 1M sucrose, 10 μM Tris-HCl, 10μl MgCl_2_, 5 μM 2-mercaptoethanol in Percoll, gradient layers were diluted with nuclear isolation buffer). The gradient was centrifuged at 7,755 RPM for 20 minutes in a Cervall HB-4 rotor, and nuclei were harvested from the interface between the two density layers. Purified nuclei were diluted in five volumes of nuclear isolation buffer and centrifuged for 10 minutes in the JA20 rotor at 7,300 RPM. The supernatant was discarded, and the pellet was resuspended in 200 μl of 1X Tris-EDTA. The DNA was purified from the nuclei using the DNeasy Plant Mini Kit (Qiagen, [www.qiagen.com](http://www.qiagen.com/)).

*S2. DNA Library Preparation and Sequencing*

Paired-end libraries were sequenced on full lanes of both the Illumina HiSeq (100 bp paired-end, insert size = 280 bp, Illumina TruSeq Adaptor #18 - CTCCGC) and the Illumina MiSeq (250 bp paired-end, insert size = 460 bp). The MiSeq sequencing sample was sequenced without using an index read as this was a uniplex run for the purposes of barcoding in the Illumina index read. Three Illumina Nextera mate pair libraries were also sequenced using the Illumina HiSeq 2000 platform (50 bp paired-end) with 2 kb (Adaptor #2 - CGATGT), 3.5 kb (Adaptor #7 - CAGATC), and 7 kb (Adaptor #19 - GTGAAA) inserts. Pacific Biosciences (PacBio RSII) Sequencing was completed at the Mayo Clinic Molecular Biology Core (Rochester, Minnesota). A genomic DNA library with insert target size of 10 kb was prepared using the Pacific Biosciences low input 10 kb library preparation protocol using the MagBead Station. This library was run on four SMRT cells with 1x120 minute movies each.

*S3. Sequencing data quality control and preprocessing*

FASTQ files from all sequencing runs were imported into CLC Genomics Workbench Version 6.5 (CLC Bio, [www.clcbio.com](http://www.clcbio.com/)). Illumina reads were subjected to quality control using the Sequencing QC Report tool. 100 bp paired-end Illumina reads were trimmed and filtered using the following parameters: Ambiguous Trim = Yes, Ambiguous Limit = 5, Quality Limit = 0.05, Use Colorspace = No, Search Reverse Sequence = Yes, Remove 5’ terminal nucleotides = Yes, Number of 5’ Terminal Nucleotides to Remove = 5, Remove 3’ terminal nucleotides = No, Discard Long Reads = No, Save Broken Pairs = No. 250 bp paired-end Illumina reads were trimmed and filtered using the following parameters: Ambiguous Trim = Yes, Ambiguous Limit = 10, Quality Limit = 0.05, Use Colorspace = No, Search Reverse Sequence = No, Remove 5’ terminal nucleotides = Yes, Number of 5’ Terminal Nucleotides to Remove = 10, Remove 3’ terminal nucleotides = No, Discard Long Reads = No, Save Broken Pairs = No. After trimming and filtering, the 250 bp paired-end reads were merged using the Merged Overlapping Pairs tool in CLC Genomics Workbench (Mismatch cost = 4, Minimum Score = 8, Gap Cost = 4, Max Unaligned = 0). Unmerged reads were saved and also used in the initial assembly. 50 bp mate-pair Illumina reads were trimmed and filtered using the following parameters: Ambiguous Trim = Yes, Ambiguous Limit = 2, Quality Limit = 0.05, Use Colorspace = No, Search Reverse Sequence = Yes, Remove 5’ terminal nucleotides = Yes, Number of 5’ Terminal Nucleotides to Remove = 2, Remove 3’ terminal nucleotides = No, Discard Long Reads = No, Save Broken Pairs = No. The mate-pair libraries were exported as FASTQ files for scaffolding as described below.

*S4. De novo genome assembly and scaffolding*

Trimmed and filtered 100 bp paired-end and 250 bp paired-end (merged and unmerged) reads were *de novo* assembled in CLC Genomics Workbench using the De Novo Assembly tool with the following parameters: Mapping Mode = Map reads to contigs (slow), Automatic Bubble Size = Yes (50), Minimum Contig Length = 200 bp, Automatic Word Size = No, Word Size = 64, Perform Scaffolding = Yes, Automatically Detect Paired Distribution = Yes, Mismatch Cost = 3, Insertion Cost = 3, Deletion Cost = 3, Length Fraction = 0.95, Similarity Fraction = 0.9. PacBio reads were used as guidance only in producing the initial assembly. Scaffolds (≥ 1,000 bp) from the initial assembly were further scaffolded using SSPACE (parameters: -x = 1, -z = 0, -k = 3, -a = 0.7, -n = 10, -T = 8, -p = 1, -o = 15, -t = 0, -m = 32, -r = 0.9) with the Illumina mate-pair and 100 bp paired-end libraries . Remaining gaps in the assembly after the mate-pair scaffolding step were filled using the Illumina 100 bp paired-end reads using GAPFILLER (parameters: -o = 2, -m = 29, -r = 0.7, -n = 10, -g = 1, -d = 50, -t = 10, -i =10). Assembly statistics of both contigs and scaffolds are listed in Table S1. Both SSPACE and GAPFILLER were run through the corresponding PERL scripts using Strawberry Perl ([http://strawberryperl.com](http://strawberryperl.com/)) on Windows. The final scaffolds were exported for annotation using the MAKER pipeline . Twelve scaffolds were subsequently removed from the assembly following the NCBI contamination screen.

*S5. Genome annotation*

An additional computer was assembled using the components listed in Supplementary File 1 to conduct genome annotation. The BioLinux 7 operating system (http://nebc.nerc.ac.uk/tools/bio-linux) was installed on the computer used for genome annotation. The MAKER annotation pipeline and dependent programs were installed on this computer for in-lab genome annotation, including repeat identification, model training, and gene prediction. Two rounds of annotation using the MAKER pipeline were completed. The first round of annotation was used to create a training set for the SNAP gene prediction software using our previously published *Thlaspi arvense* transcriptome (est2genome=1) and *Eutrema salsungineum* (formerly *Thellungiella halophila*) protein set (Thalophila_173_protein) available from www.phytozome.net (protein2genome=1) on *Thlaspi arvense* genome scaffolds over 300 kb long. Repeat masking for both the training and final annotations were identified and masked using RepeatMasker (*Arabidopsis thaliana* repeat library athrep.fasta, available from www.giriinst.org) and RepeatRunner (repeat library ‘te_proteins.fasta’ available in RepeatRunner - http://www.yandell-lab.org/software/repeatrunner.html). A SNAP HMM training file was generated for the 1,000 best gene models using the programs Fathom and Forge (part of the SNAP package). The final round of annotation was completed using this HMM training file, and resulting GFF3 files for all scaffolds were compiled using the PERL script ‘gff3merge.pl’ included in MAKER. The predicted peptide sequences from the pennycress genome annotation were extracted and compared to the Arabidopsis TAIR10 peptide database (TAIR10_pep_20110103_representitive_model_updated) using BLASTp (Expectation value = 10.0, word size = 7, mask lower case = no, filter low complexity = yes, max number of hits = 10, protein matrix and gap costs: BLOSUM62, Existence = 11, Extension = 1). RNAseq reads from the previously published de novo transcriptome assembly (Dorn et al., 2013) were used to further validate gene models. RNAseq reads from this experiment (NCBI Short Read Archive accession number SRR802670) were trimmed (Trim Adaptor = Illumina TruSeq Indexed Adaptor 3, Ambiguous Trim = Yes, Ambiguous Limit = 2, Quality Trim = Yes, Quality Limit = 0.05, Remove 5’ terminal nucleotides = Yes, Number of 5’ terminal nucleotides to remove = 10, Save broken pairs = Yes). Trimmed paired and orphan reads were mapped to the genome assembly in CLC Genomics Workbench (Mapping type = Map to gene regions only, Maximum number of hits for a read = 10, Strand specific = Both, Count paired reads as two = Yes, Reference type = Genome annotated with genes and transcripts, Global alignment = no, Automatically detect paired distances = Yes, Similarity fraction = 0.8, Length fraction = 0.8, Mismatch cost = 2, Insertion cost = 3, Deletion cost = 3).
